# The Benefits of Vitamin D Supplementation for Athletes: Better Performance and Reduced Risk of COVID-19

**DOI:** 10.3390/nu12123741

**Published:** 2020-12-04

**Authors:** William B. Grant, Henry Lahore, Michelle S. Rockwell

**Affiliations:** 1 Sunlight, Nutrition, and Health Research Center, P.O. Box 641603, San Francisco, CA 94164-1603, USA; 2 VitaminDWiki, 2289 Highland Loop, Port Townsend, WA 98368, USA; hlahore@gmail.com; 3 Department of Human Nutrition, Foods, and Exercise, Virginia Tech, Blacksburg, VA 24061, USA; msrock@vt.edu; 4 Center for Transformative Research on Health Behaviors, Fralin Biomedical Research Institute at Virginia Tech Carilion, Roanoke, VA 24016, USA

**Keywords:** athletic performance, COVID-19, acute respiratory tract infections, immunity, team sports, vitamin D, 25-hydroxyvitamin D

## Abstract

The COVID-19 pandemic is having major economic and personal consequences for collegiate and professional sports. Sporting events have been canceled or postponed, and even when baseball and basketball seasons resumed in the United States recently, no fans were in attendance. As play resumed, several players developed COVID-19, disrupting some of the schedules. A hypothesis now under scientific consideration is that taking vitamin supplements to raise serum 25-hydroxyvitamin D [25(OH)D] concentrations could quickly reduce the risk and/or severity of COVID-19. Several mechanisms have been identified through which vitamin D could reduce the risks of infection and severity, death, and long-haul effects of COVID-19: (1) inducing production of cathelicidin and defensins to reduce the survival and replication of the SARS-CoV-2 virus; (2) reducing inflammation and the production of proinflammatory cytokines and risk of the “cytokine storm” that damages the epithelial layer of the lungs, heart, vascular system, and other organs; and (3) increasing production of angiotensin-converting enzyme 2, thus limiting the amount of angiotensin II available to the virus to cause damage. Clinical trials have confirmed that vitamin D supplementation reduces risk of acute respiratory tract infections, and approximately 30 observational studies have shown that incidence, severity, and death from COVID-19 are inversely correlated with serum 25(OH)D concentrations. Vitamin D supplementation is already familiar to many athletes and sports teams because it improves athletic performance and increases playing longevity. Thus, athletes should consider vitamin D supplementation to serve as an additional means by which to reduce risk of COVID-19 and its consequences.

## 1. Introduction

The COVID-19 pandemic has had and continues to have a major impact on life and economics globally. Among people affected are athletes and sports teams at all levels of play. The Summer Olympics originally scheduled for summer 2020 in Japan are now postponed until summer 2021. Professional sports organizations have experienced delays, interruptions, and cancelations despite practicing significant measures designed to reduce COVID-19 risk. Collegiate sports seasons have been delayed, canceled, or dramatically altered by the pandemic [1,2,3]. Some programs have even been eliminated in light of growing financial challenges [4]. High school sports, amateur races, and recreational sports have been put on hold. A Google Forms survey of 692 elite and semi-elite South African athletes conducted in April found that in response to COVID-19 reductions in sports events, many of the athletes consumed excessive amounts of carbohydrates, felt depressed, and required motivation to keep active. [5]. Thus, additional methods to reduce risk of COVID-19 for athletes would be useful, especially if they might also improve athletic performance.

This narrative review outlines the use of vitamin D supplementation to raise serum 25-hydroxyvitamin D [25(OH)D] concentrations to optimal values, which may be at least 40 ng/mL for sports (e.g., [6]). The benefits of vitamin D for athletic performance and general well-being are similarly reviewed.

## 2. Results

### 2.1. Introduction to COVID-19

COVID-19 is caused by the body’s dysregulated immune response to the SARS-CoV-2 virus [7]. (macrophage activation, associated with the “cytokine storm,” promotes the dysregulation of innate immunity [8].) The virus enters largely through the lungs. The virus can enter cells by attaching to the angiotensin-converting enzyme 2 (ACE2) receptor. SARS-CoV-2’s binding to ACE2 makes more angiotensin II available to cause damage [9]. The infection also increases inflammation by ramping up production of both proinflammatory and anti-inflammatory cytokines, which can result in a cytokine storm [10]. By increasing inflammation, the cytokine storm injures the epithelial layer of the lungs—which can lead to pneumonia, acute respiratory distress syndrome (ARDS), and sepsis [7]—and later, other internal organs, which can lead to permanent damage. The T-helper 1 (Th1) and macrophage-based proinflammatory cytokines are interleukin 1β (IL-1β), IL-2, IL-6, tumor necrosis factor α, and IL-17 [7,10,11]. Approximately 70% of fatal COVID-19 cases are due to ARDS, whereas sepsis accounts for approximately 28% [7].

COVID-19 can progress through various stages [12]. The first stage is generally limited to upper respiratory tract infection accompanied by fever, fatigue, and muscle ache, whereas nausea and diarrhea are infrequent symptoms at onset [13]. The second stage is pneumonia (infection of the lower respiratory tract) with or without dyspnea (labored breathing). The third stage is complications, which could include ARDS, sepsis, cardiac and kidney injury, and secondary infection [14]. The fourth stage is death or healing. Death is unlikely for athletes because the main risk factors for death are older age [15], various chronic diseases, and elevated systemic inflammation [16].

A rapidly increasing body of research reports the benefits of vitamin D in reducing risk and severity of SARS-CoV-2 infection and COVID-19. A recent review summarized the findings as of mid-October 2020 [17]. By that time, at least 14 observational studies and a few intervention studies as well as several mechanisms related to vitamin D had been published.

### 2.2. Observational Studies of 25(OH)D and COVID-19

More than 15 observational studies of COVID-19 incidence, severity, and/or death with respect to serum 25(OH)D concentrations have been published in peer-reviewed journals. The findings in those studies are tabulated in a companion paper [17]. Although three studies reported no beneficial effect related to 25(OH)D, the others reported an inverse correlation between 25(OH)D concentrations and severity of COVID-19. Two of those studies that showed no benefit used 25(OH)D concentration values from blood drawn more than a decade before the incidence of COVID-19 and in the multivariable analysis included factors that affect 25(OH)D concentrations. In summary, mean 25(OH)D concentrations <15 ng/mL are generally associated with greater severity and risk of death for COVID-19 patients, whereas mean 25(OH)D concentrations for less severe but still hospitalized COVID-19 patients ranged from 17 to perhaps 30 ng/mL. Thus, the 10 observational studies suggest that 25(OH)D concentrations <30 ng/mL are associated with increased risk of COVID-19 infection but that the risk with respect to higher concentrations cannot be ruled out. Thus, it would be prudent to assume that higher values, such as between 40 and 60 ng/mL, might be the more appropriate range.

An observational study conducted in Chicago included 489 COVID-19 patients with a mean age of 49 ± 18 years, 75% of whom were women. Those patients presented at University of Chicago Medicine between March 3 and April 10, 2020, and had 25(OH)D concentrations measured within the past year [18]. Patients were deemed vitamin D deficient if their most recent serum 25(OH)D concentrations within 1 year before their first COVID-19 tests were <20 ng/mL for 25(OH)D or <18 pg/mL for 1,25(OH)_2_D. Patients were deemed not deficient if their most recent concentrations were ≥20 ng/mL for 25(OH)D or ≥18 pg/mL for 1,25(OH)_2_D. The relative risk of COVID-19 was 1.77 (95% confidence interval [CI], 1.12 to 2.81; *p* = 0.02) for deficient vs. non-deficient vitamin D status. The relative risk for COVID-19 for non-white vs. white race was 2.54 (95% CI, 1.26 to 5.12; *p* = 0.009).

Observational studies by themselves are not considered reliable indicators of causal relationships because confounding factors may play important roles. For 25(OH)D, sun exposure and diet [19] are two important contributing factors other than vitamin D supplementation, and they may have effects independent of vitamin D such as destroying viruses [20]. In addition, the disease state may affect 25(OH)D concentrations [21,22]. That concern is particularly important for people with chronic diseases. Thus, randomized controlled trials (RCTs) are considered the best way to determine causality.

Observational studies have also offered insight into who is at greater risk of developing COVID-19 and the severity of the disease. In the United States, African Americans and Hispanics have had much higher rates of infection and death than European Americans. [23,24,25]. In addition, people who are elderly, who are obese, and/or who have chronic conditions are at greater risk [25]. Diabetes is also an important risk factor [26]. Although African Americans and Hispanics have lower 25(OH)D concentrations than European Americans [27], many other factors help explain the incidence–severity relationships such as prevalence of other diseases and working and living in close contact with many people. A recent review outlined the evidence regarding elevated chronic disease rates for African Americans, including cardiovascular disease, diabetes, hypertension, and pulmonary disease [23]. A recent publication outlined the reasons why vitamin D deficiency in African Americans contributes to their increased risk of COVID-19 [28]. In June 2020, another publication noted the increase in COVID-19 death rates of dark-skinned Americans [29].

Seropositivity to SARS-CoV-2 is a precursor to COVID-19, which can develop after a dysregulated immune response to the virus. The correlation between SARS-CoV-2 positivity with respect to deseasonalized serum 25(OH)D concentrations measured within the past year for more than 190,000 patients by Quest Diagnostics was reported recently [30]. Non-Hispanic black people had approximately double the SARS-CoV-2 seropositivity of non-Hispanic white people over the 25(OH)D concentration range from <20 to >60 ng/mL, whereas Hispanic people had seropositivity rates approximately 60% higher than those of non-Hispanic white people. On the basis of the dependence of seropositivity on race and serum 25(OH)D concentration, researchers estimated that mean population serum 25(OH)D concentrations explained 20% of SARS-CoV-2 seropositivity among non-Hispanic black people and 30% of SARS-CoV-2 seropositivity among Hispanic people [17].

### 2.3. Treating COVID-19 with Vitamin D

The results of the first vitamin D RCT to treat COVID-19 patients were reported in late August 2020 [31]. The mean age of patients was 53 ± 11 years, and 54% of treated patients were males. Fifty were randomized to be treated with calcifediol [25(OH)D] in addition to the standard of care, whereas 26 were treated only with the standard of care. The calcifediol treatment was 0.532 mg on the day of admission and then 0.266 mg on days 3 and 7 and then weekly until discharge or admission to the intensive care unit (ICU). The conversion from calcifediol to cholecalciferol (vitamin D_3_) was given as 3.2 times the molecular weight of each; therefore, 0.532 mg of calcifediol is approximately 68,000 IU of vitamin D_3_. Calcifediol has an advantage over vitamin D_3_ in not having to go through the liver to be processed. However, as reported in the New York study, large doses of vitamin D were effective in treating COVID-19 patients [32]. Whereas only one treated patient had to enter the ICU, 13 of those given only the standard of care treatment had to do so. The univariate risk odds ratio for ICU for patients with calcifediol treatment was 0.02 (95% CI, 0.002 to 0.17).

A second vitamin D RCT to treat COVID-19 patients was reported from India [33]. COVID-19 patients admitted to a tertiary care hospital in north India were invited to the study. The criteria for participation included being mildly symptomatic or asymptomatic with or without comorbidities, that serum 25(OH)D was <20 ng/mL, and that participants were able to take oral vitamin D supplementation (e.g., not requiring invasive ventilation or with significant comorbidities). Forty patients were enrolled: 16 were randomized to receive 60,000 IU/day of vitamin D_3_ for 7 days, whereas 24 served as controls. Members of the treatment group who did not achieve a 25(OH)D concentration >50 ng/mL in the 7 days were supplemented with 60,000 IU/day for another 7 days. The mean age was ~50 years (range, 36 to 51 years). Mean 25(OH)D was 9 ng/mL (range, 7 to 13 ng/mL) in the treatment group and 19 ng/mL (range, 8 to 13 ng/mL) in the control group. Serum 25(OH)D increased by 42 ng/mL (range, 39 to 49 ng/mL) in the treatment group and 5 ng/mL (range, 0 to 12 ng/mL) in the control group. Fibrinogen decreased from 4.1 g/L (range, 3.7 to 5.1 g/L) to 3.2 g/L (range, 1.7 to 4.1 g/L) in the treatment group but was essentially unchanged in the control group: 3.7 g/L (range, 3.4 to 4.3 g/L) vs. 3.7 g/L (range, 2.4 to 4.3 g/L) (*p* = 0.001). As a result, 10 (63%) participants in the intervention group and five (22%) participants in the control arm (*p* < 0.02) became SARS-CoV-2 RNA negative.

A recent “quasi-experimental” study of high-dose vitamin D supplementation in a French nursing home shows the benefit of maintaining high 25(OH)D concentrations [34]. Sixty-three of 96 elderly residents developed COVID-19. The residents had been receiving single oral doses of 80,000 IU of vitamin D_3_ every 2–3 months. During 36 ± 17 days of follow up, 83% (57) residents who had received vitamin D within 1 month before to 1 week after diagnosis of COVID-19 compared to 44% of the nine who did not. The fully adjusted hazard ratio for survival with respect to vitamin D was 0.11 (95% CI, 0.03 to 0.48; *p* = 0.003). Those authors reported similar results for 77 consecutive COVID-19 patients in a geriatric hospital [35]. Of course, many athletes are larger than nursing home residents and so should take higher daily average vitamin D supplements. As of 9 November 2020, 30 observational studies report that COVID-19 or SARS-CoV-2 positivity was associated with lower serum 25(OH)D concentration [36]. In addition, two small-scale RCTs with vitamin D supplementation have been reported and at least 33 clinical trials have been registered [37].

### 2.4. Mechanisms of Vitamin D against SARS-CoV-2 and COVID-19

Vitamin D has several main mechanisms by which it reduces risks of COVID-19 [17,38]. One is through mounting a defense against the virus, in part through induction of cathelicidin (LL-37) and defensins. LL-37 acts at several steps in viral infection and is effective against both enveloped and non-enveloped viruses [39]. LL-37 also affects regulatory T cells. In one study, higher levels of LL-37 in serum corresponded to lower expression of IL-17 in the tonsils and to lower levels of its transcription factor, RORC2, both of which are necessary for the development of Th17 cells [40], FOXP3 (a transcription factor involved in inducing regulatory T cells) also was expressed at lower levels [41]. Several papers suggested that IL-17 was involved in the pathology of COVID-19, including risk of thrombosis [42] and ARDS [43]. A 2016 article reported that athletes who took 5000 IU/day of vitamin D_3_ for 14 weeks increased mean 25(OH)D from 22 to 50 ng/mL, resulting in a 15% increase in the concentration of cathelicidin in plasma [44].

A second mechanism is to regulate the production of cytokines, generally upregulating anti-inflammatory cytokines such as IL-10, and downregulating proinflammatory cytokines such as IL-6 [45]. Such regulation can reduce risk of the cytokine storm. An ecological study reported that influenza case-fatality rates in the United States during the 1918–1919 influenza pandemic were significantly lower in southwestern communities than in northeastern communities [46]. The mechanism proposed was vitamin D production from solar ultraviolet-B (UVB) exposure through reducing the cytokine storm.

A third mechanism is through increasing concentration of ACE2. That higher concentration counters the effect of SARS-CoV-2′s binding to the enzyme ACE2, making more angiotensin II available to cause damage [9]. In addition, increasing ACE2 may shift the balance within the renin–angiotensin–aldosterone system toward the favorable ACE2–Ang-(1-7)–MasR pathway [47]. Thus, vitamin D inhibits mediators of the renin–angiotensin–aldosterone system—present in nearly all cells of the human body—and by inhibiting ACE activity and increasing ACE2, it lowers angiotensin II levels [9] A recent article reported that ACE2 concentrations are inversely correlated with damage to heart and lung tissues [48].

The apparent role of angiotensin II in modulating or suppressing B-cell response may also become of great interest for a better understanding of the pathophysiology of coronavirus infections [49]. As discussed in a recent review, vitamin D affects B-cell activation [8], as discussed in an earlier paper [50].

In general, innate immune responses (Toll-like receptors, type I interferons, macrophages, and dendritic cells) represent the initial host defense against invading pathogens. The innate immune system inhibits virus replication, promotes virus clearance, induces tissue repair, and triggers a prolonged adaptive immune response (T cells produce proinflammatory cytokines via the NF-κB and mitogen-activated protein kinase signaling pathways) against the viruses. Pulmonary and systemic inflammatory responses associated with coronaviruses are usually triggered by the innate immune system when it recognizes the viruses [51,52].

### 2.5. COVID-19 and Athletes

Of particular concern to athletes is that COVID-19 can cause both short-term and permanent damage to many organs. Damage has been noted in the lungs [53], respiration regulation mechanisms [54], and cardiovascular system [55]. Other organs also are damaged [10]. Organ damage would reduce athletic performance. A recent review concluded that physical function and fitness are impaired following SARS-CoV-2 infection, and impairments can last for a year or more [56]. Thus, it is imperative that athletes try to reduce risk of COVID-19; supplementing with vitamin D appears to be an effective and efficient way to do so if high enough 25(OH)D concentrations are achieved.

Athletes who recover from COVID-19 may have lingering damage or other health concerns such as chronic fatigue, which could be considered a fifth stage of the disease. Lung damage is one concern [57]. More importantly, heart damage also is a concern. Damage to the heart from the cytokine storm can include decrements in heart function as well as myocarditis, acute coronary syndromes, heart failure, arrhythmias, and venous thromboembolism [58]. The clinical syndromes include acute myocardial injury, myocarditis, acute coronary syndromes, heart failure, arrhythmias, and venous thromboembolism [59]. Adverse effects can also befall the musculoskeletal, hematologic, and gastrointestinal systems [60]. Thus, athletes who have had COVID-19 should be monitored by physicians before returning to practice and competition [58].

Another concern is that because physical activity is curtailed during and shortly after COVID-19, maintenance of key physical qualities, such as game-specific contact skills and decision-making ability, are challenged, affecting performance and risk of injury on resumption of training and competition. However, strategies exist that can dramatically mitigate potential losses [61].

Several publications offer recommendations for competitive athletes returning to sports. An infographic has been prepared for graduated return-to-play guidance after COVID-19 infection [62]. Another publication proposed an algorithm for return to sports [63]. Another gave ideas to consider when fans are permitted to attend events [64]. The Australian Institute of Sport presented a framework for rebooting sport in a COVID-19 environment [65], as did the Royal Spanish Football Federation [66]. Unfortunately, those publications do not address the long-haul problem of chronic fatigue, which exercise exacerbates. Inflammatory myocarditis also has been suggested [67].

### 2.6. Other Micronutrients

Several other micronutrients have been studied regarding their impact on COVID-19 incidence and treatment, including vitamin A (retinol), vitamin C (ascorbic acid), magnesium, selenium, and zinc. Several general reviews discussed the role of micronutrients in improving the immune response to viral infections, including COVID-19 [68,69,70,71,72,73].

### 2.7. African Americans

In the United States, many collegiate and professional athletes are of African American or Hispanic race or ethnicity. As a result of dark skin pigmentation, they generally have lower 25(OH)D concentrations than European Americans. For the period 2009–2010, mean 25(OH)D concentrations determined from the National Health and Nutrition Examination Survey (NHANES) dataset were as follows: non-Hispanic black, 18 ± 2 ng/mL; Mexican American, 22 ± 1 ng/mL; non-Hispanic white, 30 ± 1 ng/mL [74]. For the period 2011–2014, the prevalence of 25(OH)D concentration <12 ng/mL in the United States for different ethnicities was as follows: non-Hispanic black, 18%; Hispanic, 6%; non-Hispanic white, 2% [75]. African Americans have stronger bones than European Americans as a result of excreting calcium at lower rates, most likely as an adaptation to life in the hot, dry environment of Africa [76]. Because the classical benefit of vitamin D relates to regulation of calcium and phosphorus absorption and metabolism, many people think that African Americans do not need to increase serum 25(OH)D concentrations. However, it is now realized that for non-skeletal effects, the role of vitamin D is essentially independent of race or ethnicity (Ames, Grant, Willett, in preparation).

African Americans and Hispanic Americans have much higher case and mortality rates of COVID-19 than European Americans or Asian Americans [77]. One reason is that they have higher rates of chronic diseases that are risk factors for COVID-19, including cardiovascular disease, diabetes mellitus, hypertension, and pulmonary disease [23]. Those diseases are associated with chronic systemic inflammation, and COVID-19 might be likely to induce production of enough additional cytokines to result in a cytokine storm [78]. Another reason for the higher disease prevalence in African Americans is their overrepresentation in high-risk broad occupational categories, such as health occupations, as well as working in low-income occupations that put them at greater risk of exposure to COVID-19 than other workers [79]. A third reason is that they have lower 25(OH)D concentrations. For the period 2001–2004, white males aged 20–39 years had mean 25(OH)D concentrations of 26 ng/mL, black men had 15 ng/mL, and Mexican American men had 22 ng/mL [27]. In the UK, “deceased doctors of Black, Asian and minority ethnic (BAME) comprised 94% of the total deaths figures in the UK, notwithstanding that they represent 44% of the workforce. The trend was similar among nurses; 71% of COVID-19 fatalities were in the BAME group, although they account for 20% of the workforce.” [80].

### 2.8. Athletic Performance

For some time, sports teams have been aware of the benefits of vitamin D supplementation to improve athletic performance. A 2009 review by Cannell and colleagues increased the interest of vitamin D among athletes [81]. It reviewed the evidence that many athletes have vitamin D deficiency, that Russian and German investigators showed improved athletic performance though UVB irradiation starting in the 1930s, that athletic performance improves after solar or artificial UVB irradiation, and that vitamin D has been shown to improve athletic performance. Interestingly, after publication of that review, the Chicago Blackhawks ice hockey team was supplemented with 5000 IU/day of vitamin D_3_ and improved from near the bottom rank in 2009 to win the Stanley Cup in 2010 [82]. Now many sports teams have their players supplement with vitamin D [83,84].

A 2020 review by de la Puente Yague and colleagues outlined the important benefits of vitamin D for athletes [85]. Table 1 presents selected findings related to those benefits.

A review of the effects of vitamin D on muscles noted that vitamin D increases the number of type II, or fast-twitch, muscle cells, including type IIA fibers, which are associated with muscular high-power output [97].

A recent study confirmed the benefit of vitamin D supplementation in maintaining optimal serum 25(OH)D concentrations after the summer season. A 12 week intervention study was conducted in which 19 college swimmers in Virginia were given 5000 IU/day of vitamin D_3_ or placebo from August to November [98]. Those in the treatment arm increased mean 25(OH)D concentration from 47 to 61 ng/mL, whereas those in the control arm had 25(OH)D decrease from 44 to 33 ng/mL. Fat-free mass increased in the treatment arm but not in the control arm. Those in the treatment arm performed better on dead-lift and vertical-jump tests than participants in the control arm.

Researchers at Virginia Tech sent questionnaires to all NCAA Division I head athletic trainers to learn about 25(OH)D testing, vitamin D supplementation, and vitamin D-related protocols and procedures [84]. Responses were received from 249 trainers (72% response rate). The 139 programs with a full-time registered dietitian or nutritionist were more likely to have a protocol in place (*p* < 0.05). A range of 25(OH)D concentration targets resulted: 20–30 ng/mL, 3%; 30–40 ng/mL, 6%; 40–50 ng/mL, 27%; >50 ng/mL, 13%; unsure, 51%. Programs that participated in the Football Bowl Subdivision were more likely to have 25(OH)D concentrations measured.

### 2.9. Observational Studies of 25(OH)D Concentrations in Athletes

Dietary sources of vitamin D such as eggs, fish, and meat do not supply enough vitamin D to affect either athletic performance or risk of COVID-19. An analysis of dietary intake for U.S. professional football players indicated that 24 defensive players were obtaining 180 ± 100 IU/day of vitamin D_3_ from dietary intake, whereas 20 offensive players were obtaining 150 ± 90 IU/day [99].

An analysis was reported for 25(OH)D concentrations in 2011 for 80 members of one U.S. football team [100]. The mean age was 27 ± 4 years and the mean 25(OH)D concentration was 27 ± 12 ng/mL. Sixty-seven players were black and 13 were white or Polynesian. Twenty-one (31%) black players had 25(OH)D <20 ng/mL, whereas no white or Polynesian players did. However, only 15 (22%) black players had 25(OH)D >32 ng/mL vs. 10 (77%) white or Polynesian players.

An analysis was reported of 25(OH)D concentrations for 33 professional football players in the National Football League ca. 2014 [101]. By race, black players had mean 25(OH)D of 27 ± 9 ng/mL, white players had 48 ± 14 ng/mL, and players of other races had 23 ± 5 ng/mL.

An observational study in Poland looked at changes of biomarkers of iron, inflammation, and vitamin D during an 8 month competitive season [102]. Among the participants, 14 players had an average of 20 ± 5 years of training plus competition; 10 non-athletes served as controls. A measure of inflammation, IL-6, increased by 77% (95% CI, 35% to 131%) between athletes and controls. Serum 25(OH)D concentrations decreased by 12% (95% CI, 20% to 3%) between athletes and controls. Systemic inflammation is an important hallmark of chronic disease [103], so taking steps to slow the increase in systemic inflammation with playing time and age, such as through vitamin D supplementation, could reduce risk of chronic diseases later in life.

A study of 25(OH)D concentrations for 105 professional ice hockey players from three teams in Canada and the United States was conducted in September 2015 [104]. The results showed 13% with insufficient 25(OH)D (<32 ng/mL), 22% with sufficient 25(OH)D (≥32 to 39.9 ng/mL), and 65% with ideal 25(OH)D concentration (≥40 ng/mL). Evidently the 2009 publication by Cannell and colleagues [81] in this journal had a lasting impact on the sport. Interestingly, the authors noted that vitamin D-sufficient players were nearly 3 years older than those who were vitamin D insufficient. The researchers suggested that the players’ higher vitamin D status enabled them to have a longer playing career.

A review of 25(OH)D concentrations, fractures, and rates of being drafted into the National Basketball Association (NBA) in round 1 or 2 was conducted for 279 athletes participating in the 2009–2013 NBA Combine [105]. The number of players in each vitamin D category were as follows: deficiency [25(OH)D = 20 ng/mL], 32%; insufficiency (20–30 ng/mL), 41%; sufficiency (>30 ng/mL), 27%. Approximately 55% of players had sustained at least one fracture, but rates were independent of 25(OH)D concentration. The rate of being drafted into the NBA increased with increasing vitamin D status: 70% for deficient, 82% for insufficient, and 85% for sufficient (*p* = 0.007).

A vitamin D supplementation study was conducted on 10 male and 10 female collegiate basketball players [106]. Five with mean baseline 25(OH)D concentration of 36 ± 6 ng/mL took 5000 IU/day of vitamin D_3_, whereas 13—11 of whom were African American, with mean baseline of 23 ± 3 ng/mL—took 10,000 IU/day. Five months later in postseason, those taking 5000 IU/day lost 4 ± 4 ng/mL, whereas those taking 10,000 IU/day gained 14 ± 11 ng/mL.

High vitamin D intake and high 25(OH)D concentrations have few adverse effects—hypercalcemia being the most severe. The symptoms of hypercalcemia may include neuropsychiatric manifestations, such as difficulty in concentration, confusion, apathy, drowsiness, depression, psychosis, and in extreme cases, a stupor and coma [107]. Only a few of the symptoms would be present in mild hypercalcemia. However, hypercalcemia seldom has serious long-term consequences if corrected, as shown in a case in which a health adviser recovered from hypercalcemia after taking 1 million IU/day of vitamin D_3_ for a month, during which his 25(OH)D concentration reached 900 ng/mL [108]. Once his 25(OH)D concentration dropped below 400 ng/mL, his hypercalcemia vanished. A study in Minnesota involving 20,308 total 25(OH)D concentration measurements over a 10 year period reported only one case of clinical toxicity associated with hypercalcemia; the concentration was 364 ng/mL [109]. One effect of high-dose vitamin D supplementation is increased absorption of calcium from the gastrointestinal tract [110]. Calcium supplementation has been linked to increased risk of myocardial infarction [111]. Thus, it is recommended that calcium supplementation be reduced when taking high-dose vitamin D.

In a meta-analysis of 48 studies with 19,833 participants in vitamin D RCTs, kidney stones were reported in only nine trials, with a tendency for fewer subjects reporting stones in the vitamin D arm than in the placebo arm (risk ratio [RR] = 0.66; 95% CI, 0.41 to 1.09; *p* = 0.10). In 37 studies, hypercalcemia was shown with increased risk shown for the vitamin D group (RR = 1.54; 95% CI, 1.09 to 2.18; *p* = 0.01). Similar increased risk of hypercalciuria was shown in 14 studies for the vitamin D group (RR = 1.64; 95% CI, 1.06 to 2.53; *p* = 0.03). [112]. However, one study used 100,000 IU/day, two studies used vitamin D_2_, and 11 studies included calcium. Eleven of the vitamin D_2_ or calcium studies had findings that supported the placebo to cause hypercalcemia, whereas only three had findings that supported vitamin D_2_ supplementation. If those 14 studies, representing 63% of the data, are omitted from the analysis, it appears very likely that the risk of hypercalcemia due to vitamin D supplementation would not be significant.

A later meta-analysis was reported by the same team, this time including studies with >2800 IU/day of vitamin D_2_ or D_3_ for a year or longer, involving 15 studies with 3150 participants [113]. “Long-term high-dose vitamin D supplementation did not increase total adverse events compared to placebo in 1731 participants from 10 studies (RR = 1.05; 95% CI = 0.88, 1.24; *p* = 0.61), nor kidney stones in 1336 participants from 5 studies (RR = 1.26; 95% CI = 0.35, 4.58; *p* = 0.72). However, there was a trend for vitamin D to increase risk of hypercalcemia in 2598 participants from 10 studies (RR = 1.93; 95% CI = 1.00, 3.73; *p* = 0.05); while its effect on hypercalciuria in only 276 participants from 3 studies was inconclusive (RR = 1.93; 95% CI = 0.83, 4.46; *p* = 0.12).” However, if one study that involved vitamin D supplementation not appropriate for athletes—100,000 IU of vitamin D_3_ per day—is omitted from the meta-analysis, the risk ratio would not have been significant.

By contrast, a psychiatric hospital in Ohio found no relationship between vitamin D and hypercalcemia: “During this time, we have admitted over 4700 patients, the vast majority of whom agreed to supplementation with either 5000 or 10,000 IUs/day. Due to disease concerns, a few agreed to larger amounts, ranging from 20,000 to 50,000 IUs/day. There have been no cases of vitamin D_3_ induced hypercalcemia or any adverse events attributable to vitamin D_3_ supplementation in any patient” [114].

### 2.10. Other Health Benefits of Vitamin D

For people likely to be athletes from their teenage years to their mid-30s, several health outcomes that may be affected by vitamin D status are of interest. Table 2 lists some of those outcomes along with the evidence for beneficial effects of vitamin D.

## 3. Discussion

Debate is ongoing regarding the advisability of measuring serum 25(OH)D concentrations. The benefits include that such measurements can help guide vitamin D supplementation doses [119]. Many factors affect the relationship between vitamin D dose and serum 25(OH)D concentration, including body mass, genetics related to absorption of vitamin D from the gastrointestinal tract, conversion from vitamin D to 25(OH)D, and baseline 25(OH)D concentrations. On the negative side is the cost and time required. In the past few years, mail-in blood spot tests have been developed that are inexpensive, convenient, and accurate [120].

Government agencies and disease organizations offer many recommendations regarding vitamin D supplementation and 25(OH)D concentrations. Two better known ones are from the U.S. Institute of Medicine [121] and the U.S. Endocrine Society [122]. The Institute of Medicine recommendations were based on requirements for bone health, recommending 600 IU/day up to age 70 years and 800 IU/day for people older than 70 years, with 20 ng/mL considered an adequate concentration. The Endocrine Society recommendation was for patients, advising 1000–2000 up to 4000 IU/day of vitamin D supplementation, with 30 ng/mL considered sufficient. The consensus statement from a vitamin D conference held in Warsaw, Poland, in 2017 stated: “The bone-centric guidelines recommend a target 25(OH)D concentration of 20ng/mL (50nmol/L), and age-dependent daily vitamin D doses of 400-800IU. The guidelines focused on pleiotropic effects of vitamin D recommend a target 25(OH)D concentration of 30 ng/mL (75 nmol/L), and age-, body weight-, disease-status, and ethnicity dependent vitamin D doses ranging between 400 and 2000 IU/day.” [123]. However, another analysis “estimated, for example, that doses of 1885, 2802 and 6235 IU per day are required for normal weight, overweight and obese individuals respectively to achieve natural 25(OH)D concentrations (defined as 23 to 68 ng/mL).” [124].

Most of the action of vitamin D is due to the hormonal metabolite, 1,25(OH)_2_D, entering vitamin D receptors attached to chromosomes, thereby affecting gene expression. A study was conducted involving “30 healthy adults randomized to receive 600, 4,000 or 10,000 IU/day of vitamin D_3_ for 6 months. Circulating parathyroid hormone (PTH), 25(OH)D, calcium and peripheral white blood cells broad gene expression were evaluated. We observed a dose-dependent increase in 25(OH)D concentrations, decreased PTH and no change in serum calcium. A plateau in PTH concentrations was achieved at 16 weeks in the 4000 and 10,000 IU/day groups. There was a dose-dependent 25(OH)D alteration in broad gene expression with 162, 320 and 1289 genes up- or down-regulated in their white blood cells, respectively.” [125]. That finding offers additional justification for 10,000 IU/day of vitamin D_3_ for athletes.

A review from Italy took a more cautionary view of vitamin D supplementation by athletes. The review noted that vitamin D can confer several benefits, including reduced risk of cancer, better brain health, improved immune system and reduced inflammation, and better muscle function by decreasing oxidative stress and supporting mitochondrial function. However, those authors also noted that some athletes take high doses to improve performance but run the risk of vitamin D toxicity manifested as hypercalcemia [126]. Also mentioned was the increased risk of prostate and pancreatic cancer at high levels of 1α,25(OH)_2_D. Findings of observational studies do indicate that mild prostate cancer incidence rates increase with increasing 25(OH)D concentrations [127]. However, prostate cancer mortality rates decrease with increasing 25(OH)D concentrations [128]. What appears to explain that dichotomy is that the classic role of vitamin D is to regulate absorption of calcium and phosphorus from the gastrointestinal tract and that calcium and phosphorus concentrations are associated with prostate cancer risk [129]. High calcium intake is a risk factor for aggressive prostate cancer for African Americans but not European Americans [130].

Of course, athletes also require other nutrients for optimal health and performance. A 2018 review discusses which nutrients might need to be supplemented for athletes [131].

Vitamin D comes in two forms, cholecalciferol (vitamin D_3_) and ergocalciferol (vitamin D_2_). Cholecalciferol is made by animals, whereas ergocalciferol is made by fungi, including yeast. In general, cholecalciferol is considered better than ergocalciferol, in part because it raises serum 25(OH)D concentration for longer and in part because it is more likely to produce beneficial health outcomes. A meta-analysis of 52 trials with a total of 75,454 participants reported that all-cause mortality rates in trials with vitamin D_3_ were significantly lower (RR = 0.95; 95% CI, 0.90 to 1.00), whereas those with vitamin D_2_ had an increased risk (RR = 1.03; 95% CI, 0.98 to 1.09). A systematic review of vitamin D supplementation regarding muscle strength in athletes indicated that vitamin D_3_ had a positive impact on muscle strength [132].

Diet also affects serum 25(OH)D concentrations. A study conducted in England showed that meat eaters and fish eaters had much higher 25(OH)D than vegetarians and vegans: mean 25(OH)D concentrations were 30, 29, 26, and 22 ng/mL, respectively [19]. Animal products such as eggs, fish, and meat can have vitamin D as both vitamin D_3_ and 25(OH)D_3_ [133]. However, because food frequency tables generally do not include the contribution of vitamin D from 25(OH)D, it is generally overlooked in dietary intake studies.

## 4. Conclusions

Athletes and people associated with them could benefit from better athletic performance, better health, and reduced risk for COVID-19 by maintaining serum 25(OH)D concentrations above 40 ng/mL. To achieve that concentration could take supplementation of vitamin D_3_ at perhaps 4000–10,000 IU/day depending on body size, skin pigmentation, and other personal factors. The 10,000 IU/day dosing level will yield a good serum concentration of vitamin D in several months. If a high concentration is desired sooner for sports performance or to avoid COVID-19, a person should consider starting with a bolus dose.

Vitamin D supplementation can be useful in reducing risk of COVID-19 and its severity, but it should not be the only measure employed. Athletes should also follow official guidelines such as regarding wearing masks, social distancing, and periodic testing.

## Figures and Tables

**Table 1:** Benefits of higher vitamin D status for athletes.

Benefit	Population	Intervention	Results	Reference
Muscle strength	163 healthy athletes	Vitamin D_3_ supplementation (5000 IU/day) in RCTs, meta-analysis	No significant effect *	[86]
Muscle strength	22 adult male white national-level judoka athletes	Bolus dose of 150,000 IU vitamin D_3_	25(OH)D concentration increased from 13 to 17 ng/mL and muscle strength by 13% in 8 days	[87]
Muscle strength and power	25 Polish elite judoists	Observational study, 25(OH)D ranged from 8 to 30 ng/mL	Left hand grip, total work during extension of the right and left lower limb, and muscle power increased by 20–30% (*r* = 0.22 to 0.32)	[88]
Muscle repair	14 recreationally active adults	Intense leg exercise of one leg	Serum 25(OH)D concentrations inversely predicted (*p* < 0.05) muscular weakness (i.e., control leg vs. exercise leg peak isometric force) immediately and days (i.e., 48 and 72 h)	[89]
Muscle repair	30 reportedly healthy and modestly active adult males (31 ± 5 years, 31 ± 8 ng/mL)	Randomly assigned to 4000 IU/day of vitamin D_3_ or placebo for 28 days and then subjected to a one-legged exercise routine	Supplemental vitamin D increased serum 25(OH)D concentrations (*p* < 0.05; ≈70%) and enhanced recovery in peak isometric force after the damaging event (*p* < 0.05; ≈8% at 24 h). Supplemental vitamin D attenuated (*p* < 0.05) immediate and delayed (48, 72, or 168 h) increase in circulating biomarkers representative of muscle damage (ALT or AST) without ameliorating muscle soreness (*p* > 0.05)	[90]
Muscle repair	20 males with baseline 25(OH)D = 18 ± 10 ng/mL	Participants performed knee-damaging exercise, supplements with 4000 IU/day of vitamin D_3_ or placebo	Supplemental vitamin D_3_ increased serum 25(OH)D and improved recovery of peak torque at 48 h and 7 days postexercise	[91]
Stress fractures	118 NCAA Division I athletes in South Carolina	Vitamin D supplementation in winter	Stress fracture rate dropped from 7.5% to 1.7% (*p* = 0.009)	[92]
Lung function	28 active college-age males, Gdansk, Poland	6000 IU/day of vitamin D_3_ for 8 weeks or placebo, January to March; mean 25(OH)D increased from 20 to 60 ng/mL	Significant improvements in maximal aerobic and anaerobic power; VO_2max_ test, maximal lung minute ventilation (VE_max_ mL·min^−1^), maximal breath frequency (BF_max_ 1·min^−1^) improved significantly	[93]
Immune function	225 endurance athletes in winter, UK	Observational study	A significantly higher proportion of subjects presented with symptoms of URTI in the vitamin D-deficient status group (initial plasma 25(OH)D < 12 ng/mL) than in the optimal vitamin D group (>48 ng/mL); total number of URTI symptom days and median symptom-severity score in vitamin D-deficient group was significantly higher	[6]
Heart size	521 male national-level athletes in Qatari; 244 lightly exercising controls	Observational study with respect to serum 25(OH)D concentration	Severely 25(OH)D-deficient athletes (25(OH)D < 10 ng/mL) present significantly smaller cardiac structural parameters than insufficient and sufficient athletes; athletes had larger cardiac parameters than controls	[94]
Traumatic brain injury	Three patients, aged 17, 23, and 31 years	Case series treated with vitamin D, progesterone, omega-3 fatty acids, and glutamine	Reversed coma and improved clinical outcomes	[95]
Stress fractures	600 navy servicewomen diagnosed with stress fracture of the tibia or fibula and 600 controls matched by race, length of service, day of blood draw	Observational study with respect to serum 25(OH)D concentration	OR for stress fracture for high 25(OH)D quintile (mean = 50 ng/mL) vs. low quintile (mean = 14 ng/mL) = 0.51 (95% CI, 0.34 to 0.76, *p* < 0.01)	[96]

25(OH)D, 25-hydroxyvitamin D; 95% CI, 95% confidence interval; ALT, alanine aminotransferase; AST, aspartate aminotransferase; BF_max_, maximal breath frequency; *r*, regression coefficient; OR, odds ratio; RCT, randomized controlled trial; URTI, upper respiratory tract infection; VE, maximum minute ventilation; VO_2max_, maximal oxygen uptake. * Trial lasted only 4 weeks and got half the athletes to levels >31 ng—enough for strength to start to increase.

**Table 2:** Evidence for beneficial effects of vitamin D for selected outcomes.

Outcome	Population	Intervention	Results	Reference
Progression to diabetes mellitus	2423 participants with prediabetes	Randomized to receive 4000 IU/day of vitamin D_3_ or placebo, 24 month duration	Various groups had reduced progression to diabetes mellitus in the secondary analyses	[115]
Progression to diabetes mellitus	2423 participants with prediabetes	Randomized to receive 4000 IU/day of vitamin D_3_ or placebo, 24 month duration	Hazard ratio for diabetes for an increase of 10 ng/mL in intratrial 25(OH)D level was 0.75 (95% CI, 0.68 to 0.82) among those assigned to vitamin D and 0.90 (95% CI, 0.80 to 1.02) among those assigned to placebo	[116]
Acute respiratory tract infection	25 eligible RCTs; IPD obtained for 10,933 of 11,321 participants	Vitamin D supplementation	Protective effects seen in individuals receiving daily or weekly vitamin D without additional bolus doses (aOR = 0.81; 95% CI, 0.72 to 0.91)	[117]
Pregnancy and birth outcomes	Pregnant women	Review	Having 25(OH)D concentration >40 ng/mL during pregnancy has many important benefits for both mother and fetus/infant	[118]

95% CI, 95% confidence interval; aOR, adjusted odds ratio; IPD, individual participant data; RCT, randomized controlled trial.
